# Acknowledgement to Reviewers of the *Journal of Functional Biomaterials* in 2014

**DOI:** 10.3390/jfb6010014

**Published:** 2015-01-09

**Authors:** JFB Editorial Office

**Affiliations:** MDPI AG, Klybeckstrasse 64, CH-4057 Basel, Switzerland

The editors of the *Journal of Functional Biomaterials* would like to express their sincere gratitude to the following reviewers for assessing manuscripts in 2014:

Alvarez-Lorenzo, Carmen
Bäumler, Hans
Bencharit, Sompop
Berron, Brad J.
Cairrão, Elisa
Caramella, Carla
Dixon, David A.
Do, Sun Hee
Dziubla, Thomas
Frisch, Benoit
Gentile, Piergiorgio
Georgieva, Radostina
Gloria, Antonio
Godin, Biana
Gotor-Fernández, Vicente
Harkin, Damien
Hashimotoa, Takashi
Hernandez-Montelongo, J.
Ho, Sunita P.
Hong, Yi
Huang, Wei Min
Hutmacher, Dietmar
Isbister, James P.
Jameson, Geoff
Jayakumar, Rangasamy
Jonkheijm, Pascal
Kerkhoff, Claus
Kietzmann, Thomas
Kwon, Il-Keun
Lammi, Mikko
Leipzig, Nic D.
Linninger, Andreas A.
Liu, Johnson
Lou, Ching-Wen
Malde, Alpesh
Matsusaki, Michiya
Mehta, Geeta
Morales, Juan C.
Müller, W. E. G.
Nagamine, Takeaki
Naviglio, Silvio
Parenteau-Bareil, Rémi
Peyton, Shelly
Pittman, Roland
Qian, Mingxing
Reale, Marcella
Rimondini, Lia
Romani, Andrea M. P.
Sakai, Hiromi
Serda, Rita
Shigdar, Sarah
Simoni, Jan
Singh, Arvind
Song, Jinhua
Soria, Bernat
Ullah, Aman
Upadhyay, Arun Kumar
Velasco Ortega, Eugenio
Vergara, Alessandro
Wang, Qun
Wennerber, Ann
Woo, Savio L-Y.
Yager, Dorne
Yoon, Eui-Sung
Yuan, Hanna S.
Zelikin, Alex
Ziebarth, Noël M.

